# A comprehensive understanding of ambient particulate matter and its components on the adverse health effects based from epidemiological and laboratory evidence

**DOI:** 10.1186/s12989-022-00507-5

**Published:** 2022-11-29

**Authors:** Tianyu Li, Yang Yu, Zhiwei Sun, Junchao Duan

**Affiliations:** 1 Department of Toxicology and Sanitary Chemistry, School of Public Health, Capital Medical University, Beijing, 100069 People’s Republic of China; 2 Beijing Key Laboratory of Environmental Toxicology, Capital Medical University, Beijing, 100069 People’s Republic of China

**Keywords:** Air pollution, Particulate matter, Environment, Health effect, Adverse outcome pathway

## Abstract

The impacts of air pollution on public health have become a great concern worldwide. Ambient particulate matter (PM) is a major air pollution that comprises a heterogeneous mixture of different particle sizes and chemical components. The chemical composition and physicochemical properties of PM change with space and time, which may cause different impairments. However, the mechanisms of the adverse effects of PM on various systems have not been fully elucidated and systematically integrated. The Adverse Outcome Pathway (AOP) framework was used to comprehensively illustrate the molecular mechanism of adverse effects of PM and its components, so as to clarify the causal mechanistic relationships of PM-triggered toxicity on various systems. The main conclusions and new insights of the correlation between public health and PM were discussed, especially at low concentrations, which points out the direction for further research in the future. With the deepening of the study on its toxicity mechanism, it was found that PM can still induce adverse health effects with low-dose exposure. And the recommended Air Quality Guideline level of PM_2.5_ was adjusted to 5 μg/m^3^ by World Health Organization, which meant that deeper and more complex mechanisms needed to be explored. Traditionally, oxidative stress, inflammation, autophagy and apoptosis were considered the main mechanisms of harmful effects of PM. However, recent studies have identified several emerging mechanisms involved in the toxicity of PM, including pyroptosis, ferroptosis and epigenetic modifications. This review summarized the comprehensive evidence on the health effects of PM and the chemical components of it, as well as the combined toxicity of PM with other air pollutants. Based on the AOP Wiki and the mechanisms of PM-induced toxicity at different levels, we first constructed the PM-related AOP frameworks on various systems.

**Graphical Abstract:**

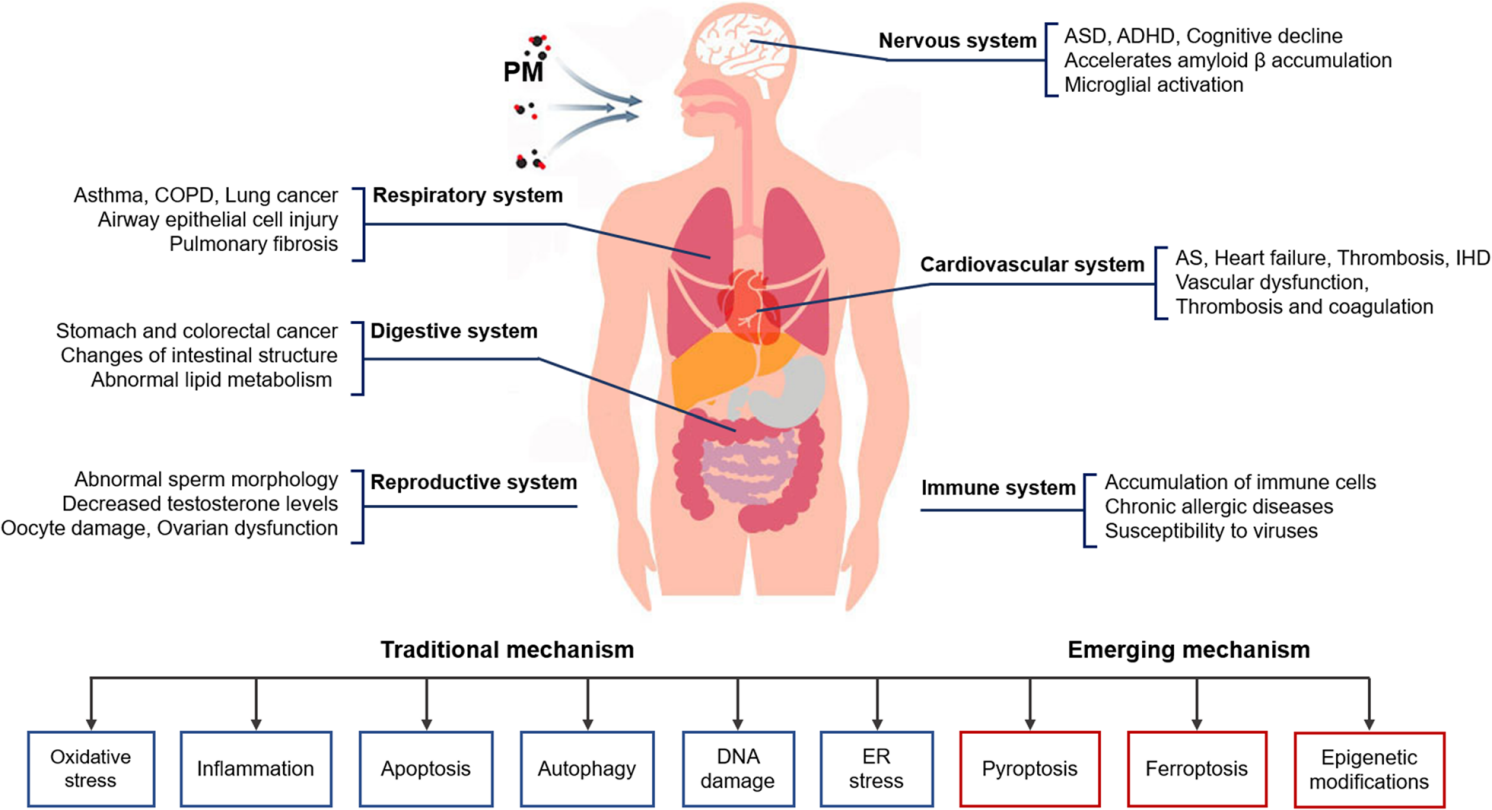

## Introduction

With the development of industry and transportation, air pollution has caused many public health events in various parts of the world. As the fourth leading risk attributable to Disability-adjusted life years (DALYs), air pollution caused 6.7 million premature deaths in 2019 [1]. According to the State of Global Air 2020, PM_2.5_ is the largest driver of the burden of disease worldwide caused by air pollution. Chronic exposure to PM_2.5_ resulted in 4.14 million deaths and 118 million lost DALYs, accounting for 62% of all deaths and 55% of DALYs due to air pollution, respectively [2]. Notably, the level of PM pollution, as the seventh leading risk attributable to Disability-adjusted life years (DALYs), increased significantly in 2019 [3]. Moreover, the PM pollution burden was 44.6% higher in Global Burden of Disease (GBD) 2019 than in GBD 2017 [4]. According to aerodynamic diameter, PM can be generally divided into PM_10_ (< 10 μm), PM_2.5_ (< 2.5 μm) and ultrafine particles (UFPs, 1–100 nm) [5]. PM contains many toxic components, among which water-soluble extracts are mainly composed of metals with high solubility/bioavailability and polycyclic aromatic hydrocarbons (PAH) with a small number of rings (Cd, Se, Ca, Sr, Rb, Zn, Mo, K, Cs and As; NAP, PYR, FLU and BaA), the organic extract is mainly composed of PAH with high oxidation potential (CHR, BPE, IPY, BbF, DBA, PHE, BkF and BaP) and the carbon core component is mainly composed of metals with low solubility/bioavailability (Mn, Fe, Na, Ni, Pb, Cr, Ba, Cu, Ti, Al and V) [6]. PM_2.5_ is the main type of particulate matter pollution [7]. Due to its small particle size, PM_2.5_ could float in the air for a long time, entering and depositing in the lung when breathing. The deposited PM_2.5_ could enter the circulatory system through the blood barrier, then reach and act on various organs and systems with the bloodstream, causing health damage [8–11].

Numerous studies have shown that PM can induce acute and chronic impacts on human health and cause harm to different systems and organs, including respiratory system, cardiovascular system, digestive system, nervous system, immune system and reproductive system [12–16]. A prospective mortality study found that long-term exposure to PM_2.5_ was a crucial risk factor for the mortality of lung cancer and cardiopulmonary disease. For every 10 μg/m^3^ increase of PM_2.5_, the risk of mortality of lung cancer and cardiopulmonary disease was elevated by 8% and 6%, respectively [17]. Besides, a study on manganese mining workers suggested that PM_2.5_ was correlated with pulmonary dysfunction, with the main impairment being restrictive ventilatory disorder, manifested by a significant decrease of peak expiratory flow rate (PEFR), the percentage of peak expiratory flow out of the overall expiratory flow volume (PEFR%), maximum mid-expiratory flow (MMEF) and forced expiratory volume per second (FEV1.0) [18]. In addition, one study confirmed the correlation between the oxidation potential of PM components such as organic carbon, elemental carbon and heavy metals with the adverse effects on human health [19]. With the deepening of the research on the toxicity mechanism of PM, previous understandings of it have been updated. Low-concentration PM_2.5_ exposure was still found to have adverse effects on human health. A study of the Medicare population in New England, which restricted the long-term exposure concentration of PM_2.5_ to annual average < 10 μg/m^3^, suggested that low-concentration PM_2.5_ exposure was associated with all-cause mortality and a 2.14% increase in mortality for every 10 μg/m^3^ increase of PM_2.5_ [20]. Besides, Shin et al. observed that PM_2.5_ exposure at an annual average of 9.8 μg/m^3^ was still correlated with the increased risk of atrial fibrillation and stroke [21]. Furthermore, the recommended annual Air Quality Guidelines (AQG) levels of PM_2.5_ and PM_10_ were respectively adjusted down to 5 μg/m^3^ and 15 μg/m^3^ in the latest Global Air Quality Guidelines released by WHO in 2021, which means that there is still great potential to determine the exposure thresholds of PM and to study the underlying mechanisms of it at low concentrations.

Previous reviews have preliminary illustrated relevant mechanisms from the perspective of PM-related cell death patterns or the perspective of certain diseases or systems, pointing out that oxidative stress, inflammation, apoptosis, autophagy and DNA damage are the main mechanisms leading to the adverse effects of PM [12, 22–24]. In vitro and in vivo studies have shown that PM_2.5_ causes oxidative stress through the induction of reactive oxygen species (ROS), which leads to dysregulation of calcium homeostasis and cytotoxicity [25, 26]. PM_2.5_ and its components can induce systemic inflammation by activating inflammation-related pathways and increasing the secretion level of inflammatory cytokines, ultimately leading to the dysfunction of various organs [27–30]. Besides, oxidative stress induced by long-term exposure to PM can lead to apoptosis and increase the expression of apoptosis-related proteins, which is an important mechanism of the adverse effects caused by PM [31, 32]. Additionally, it has been reported that PM_2.5_ led to autophagy by activating related signaling pathways, such as activation of AMP-activated protein kinase (AMPK)-induced macroautophagy and activation of PTEN-induced kinase 1 (PINK1)/Parkin pathway-driven mitophagy [33–35]. In recent years, several emerging mechanisms have been identified as being involved in the toxicity of PM, including pyroptosis, ferroptosis and epigenetic modifications. PM_2.5_ triggers lung injury and inflammation by inducing pyroptosis in lung macrophages [36]. Exposure to PM_2.5_ can induce the destruction of iron and redox balance in endothelial cells, resulting in ferroptosis and the secretion of inflammatory cytokines [37]. Additionally, epigenetic changes such as histone modification and DNA methylation have been found to be involved in the health damage caused by PM [38–40]. However, a framework linking molecular or cellular events to PM-related adverse outcomes in animal models, individuals or populations is still lacking, which can facilitate comprehensive elucidation of the mechanisms of PM-induced toxicity on various systems.

**Fig. 1:**
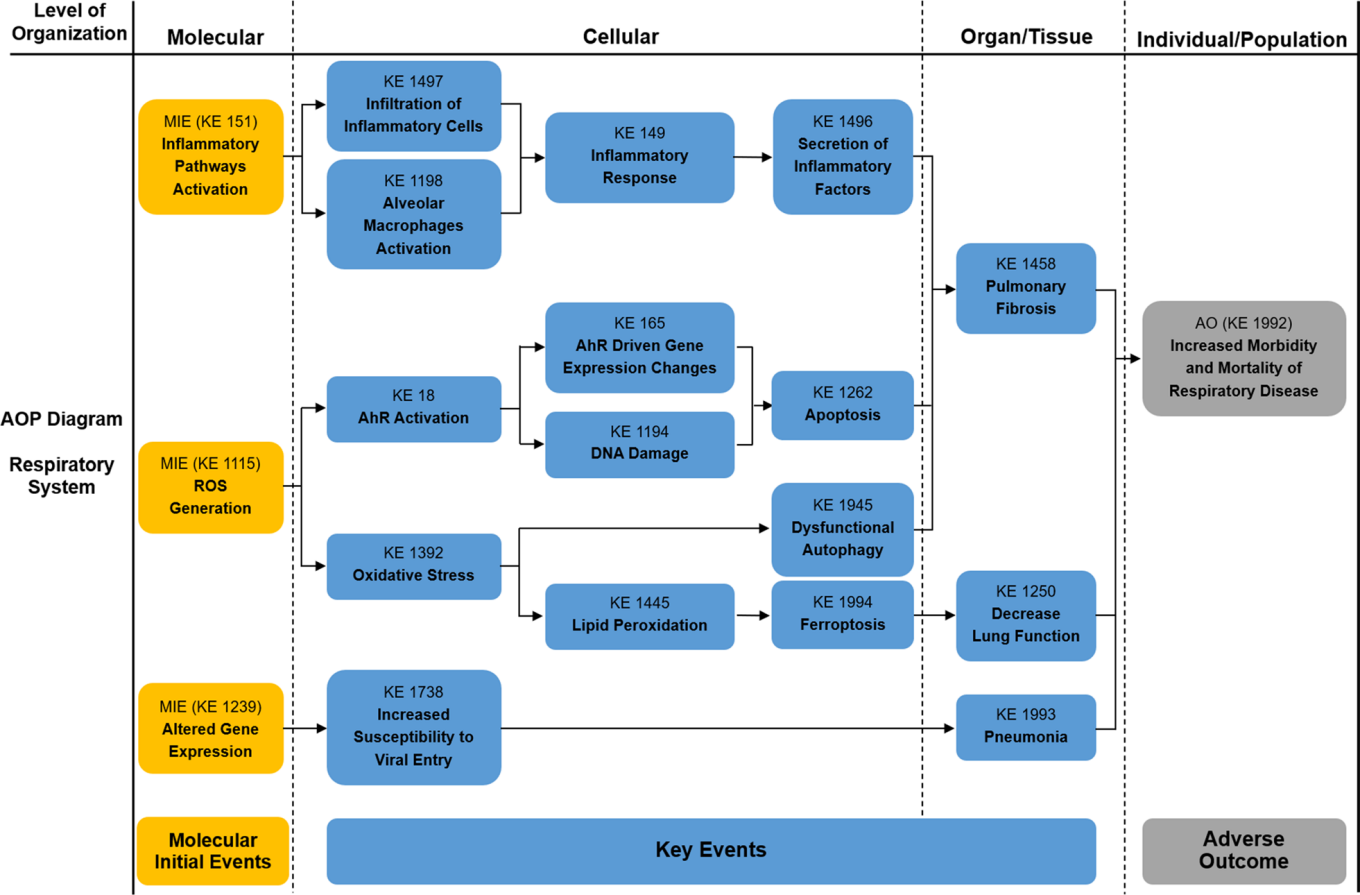
Adverse Outcome Pathways diagram related to PM associated respiratory toxicity. Orange cuboid: Molecular Initial Events (MIE); Blue cuboid: Key Events (KE); Gray cuboid: Adverse Outcome (AO)

**Fig. 2:**
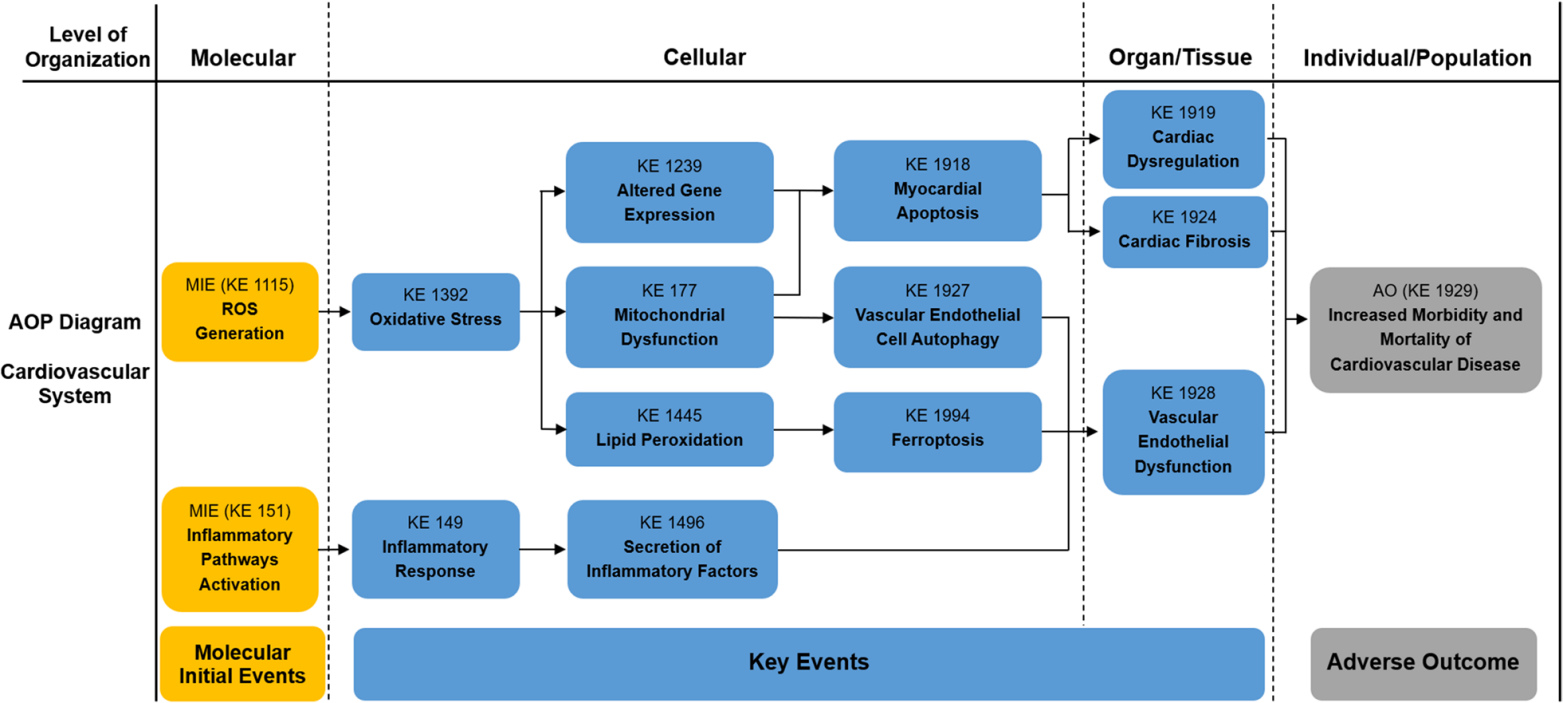
Adverse Outcome Pathways diagram related to PM associated cardiovascular toxicity. Orange cuboid: Molecular Initial Events (MIE); Blue cuboid: Key Events (KE); Gray cuboid: Adverse Outcome (AO)

**Fig. 3:**
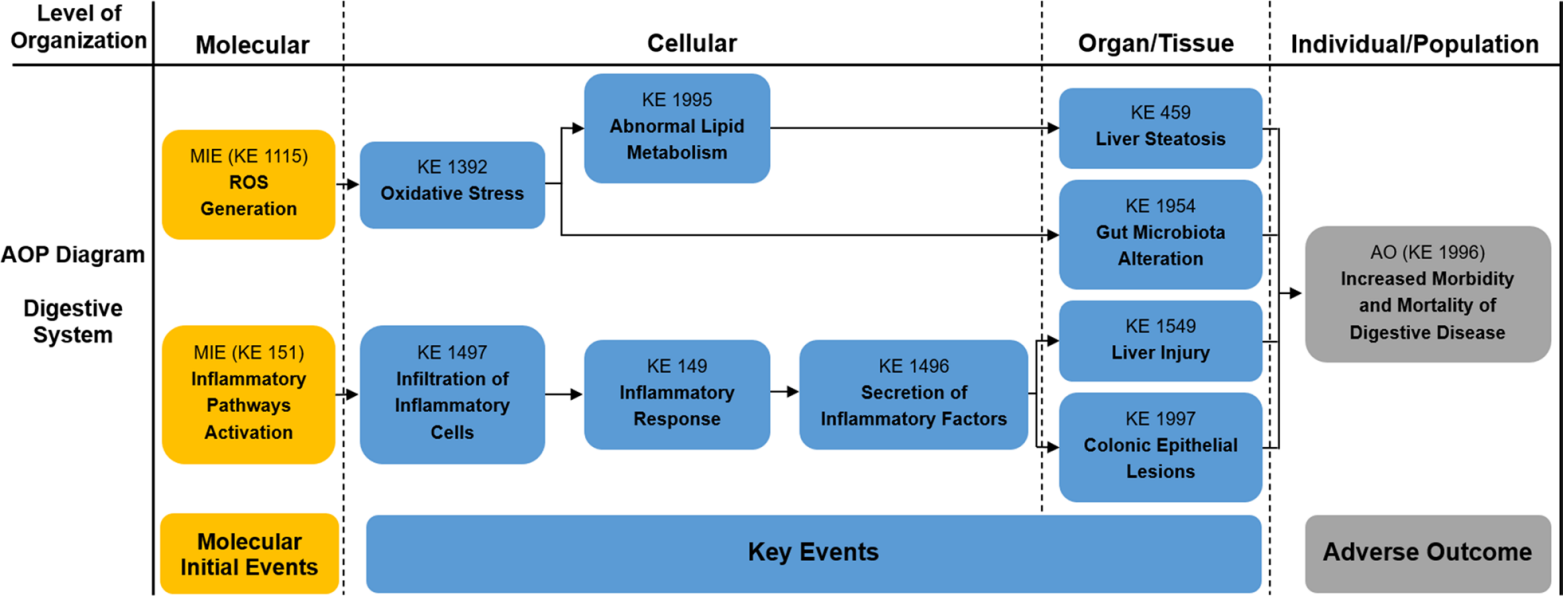
Adverse Outcome Pathways diagram related to PM associated digestive toxicity. Orange cuboid: Molecular Initial Events (MIE); Blue cuboid: Key Events (KE); Gray cuboid: Adverse Outcome (AO)

**Fig. 4:**
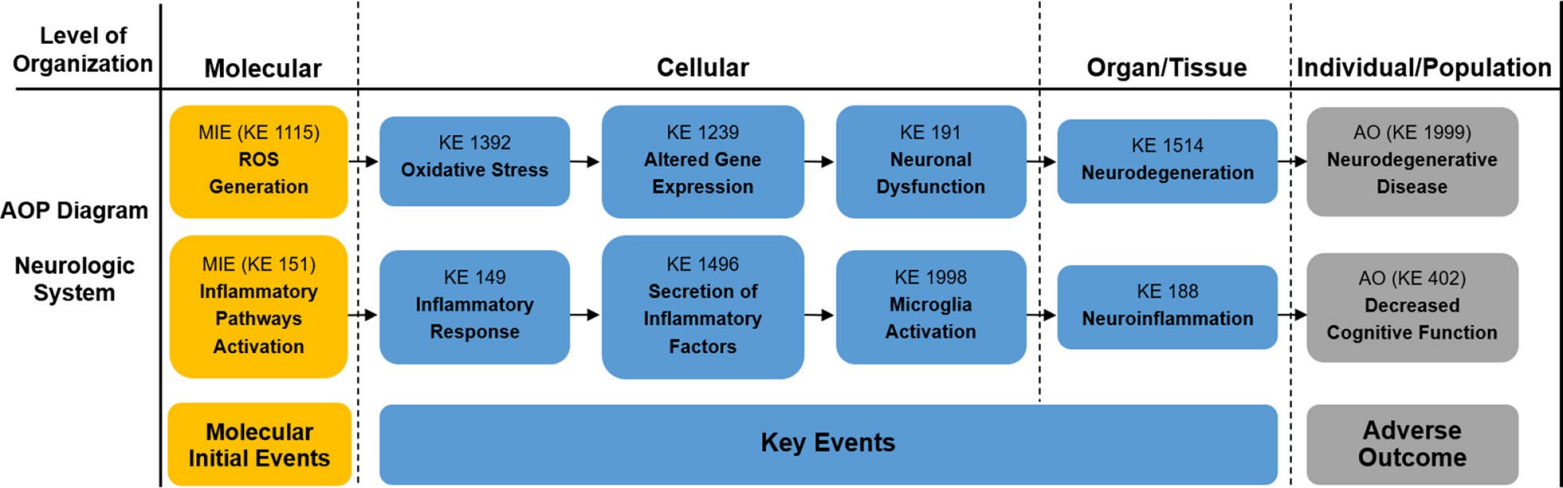
Adverse Outcome Pathways diagram related to PM associated neurologic toxicity. Orange cuboid: Molecular Initial Events (MIE); Blue cuboid: Key Events (KE); Gray cuboid: Adverse Outcome (AO)

**Fig. 5:**
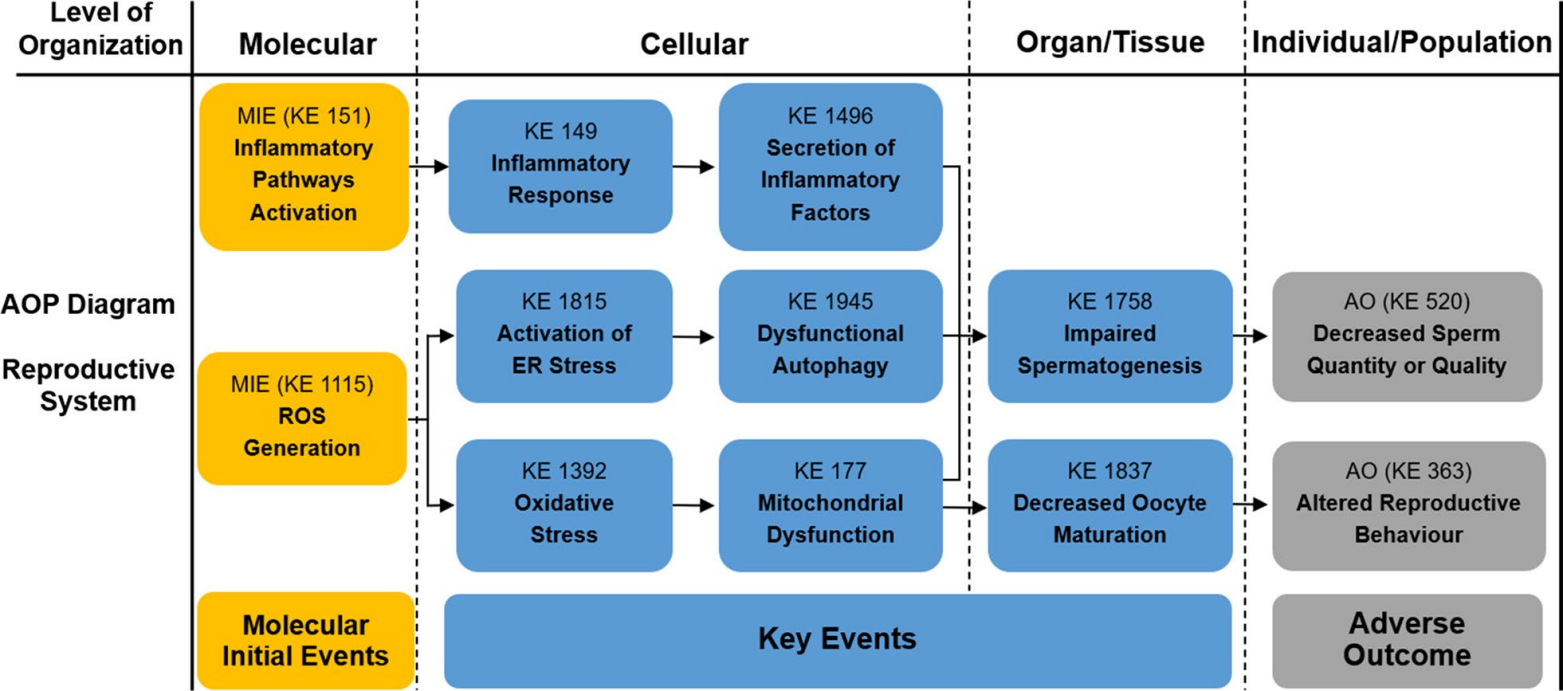
Adverse Outcome Pathways diagram related to PM associated reproductive toxicity. Orange cuboid: Molecular Initial Events (MIE); Blue cuboid: Key Events (KE); Gray cuboid: Adverse Outcome (AO)

As described in our previous study, Adverse Outcome Pathway (AOP) is a conceptual framework for organizing, synthesizing, and presenting the linkages between disturbance of the molecular initiating event (MIE), key events (KEs) and adverse outcome (AO) by a stressor [41]. The core content of AOP is to standardize and modularize a series of toxic events from molecule, cell, organ to individual and population, as well as emphasize determining the relationship between these toxic events by logical reasoning. In recent years, the AOP framework has been more and more applied to the risk assessment of environmental pollution and chemical exposure, making it possible to develop risk management strategies based on the mechanism of toxicity [42]. In our previous study, an AOP framework focused on PM_2.5_-induced cardiovascular toxicity was proposed [41]. Based on the AOP Wiki and the mechanisms of PM-induced toxicity at different levels, we first constructed the PM-related AOP frameworks on various systems, as shown in Figs. 1, 2, 3, 4, 5. The MIEs, KEs and AOs of various systems were summarized in Tables 1, 2, 3, 4, 5.

**Table 1:** Summary of the AOP of respiratory system

Sequence	Type	Event ID	Title	Short name
1	MIE	1115	ROS generation	Increased, Reactive oxygen species
2	MIE	151	Inflammatory pathways activation	Inflammatory pathways activation
3	MIE	1239	Altered gene expression	Altered, Gene Expression
4	KE	1392	Oxidative stress	Oxidative stress
5	KE	1945	Dysfunctional autophagy	Dysfunctional autophagy
6	KE	18	AhR activation	Activation, AhR
7	KE	165	AHR driven gene expression changes	AHR driven gene expression changes
8	KE	1194	DNA damage	DNA damage
9	KE	1445	lipid peroxidation	Increase, LPO
10	KE	1262	Apoptosis	Apoptosis
12	KE	1994	Increase, Ferroptosis	Ferroptosis
13	KE	1497	Infiltration of inflammatory cells	Infiltration of inflammatory cells
14	KE	1198	Activity of alveolar macrophages	Activation, Macrophages
15	KE	149	Inflammatory response	Increase, Inflammation
16	KE	1496	Secretion of inflammatory factors	Increased proinflammatory mediators
17	KE	1738	Increased susceptibility to viral entry	Increased susceptibility to viral entry
18	KE	1458	Pulmonary fibrosis	Pulmonary fibrosis
19	KE	1250	Decrease Lung function	Decrease, Lung function
20	KE	1993	Pneumonia	Pneumonia
21	AO	1992	Increased morbidity and mortality of respiratory disease	Increased morbidity and mortality of respiratory disease

*MIE* molecular initiating events, *KE* key events, *AO* adverse outcomes

**Table 2:** Summary of the AOP of cardiovascular system

Sequence	Type	Event ID	Title	Short name
1	MIE	1115	ROS generation	Increased, Reactive oxygen species
2	MIE	151	Inflammatory pathways Activation	Inflammatory pathways Activation
3	KE	1392	Oxidative stress	Oxidative stress
4	KE	1239	Altered gene expression	Altered, Gene Expression
5	KE	177	Mitochondrial dysfunction	Mitochondrial dysfunction
6	KE	1445	lipid peroxidation	Increase, LPO
7	KE	1918	Myocardial apoptosis	Myocardial apoptosis
8	KE	1927	Vascular endothelial cell autophagy dysfunction	Vascular endothelial cell autophagy dysfunction
9	KE	1994	Increase, Ferroptosis	Ferroptosis
10	KE	149	Inflammatory response	Increase, Inflammation
11	KE	1496	Secretion of inflammatory factors	Increased proinflammatory mediators
12	KE	1919	Cardiac dysregulation	Cardiac dysfunction
13	KE	1924	Cardiac fibrosis	Cardiac fibrosis
14	KE	1928	Vascular endothelial dysfunction	Vascular endothelial dysfunction
15	AO	1929	Increased morbidity and mortality of cardiovascular disease	Increased morbidity and mortality of cardiovascular disease

*MIE* molecular initiating events, *KE* key events, *AO* adverse outcomes

**Table 3:** Summary of the AOP of digestive system

Sequence	Type	Event ID	Title	Short name
1	MIE	1115	ROS generation	Increased, Reactive oxygen species
2	MIE	151	Inflammatory pathways Activation	Inflammatory pathways Activation
3	KE	1392	Oxidative stress	Oxidative stress
4	KE	1995	Abnormal lipid metabolism	Abnormal lipid metabolism
5	KE	1497	Infiltration of inflammatory cells	Infiltration of inflammatory cells
6	KE	149	Inflammatory response	Increase, Inflammation
7	KE	1496	Secretion of inflammatory factors	Increased proinflammatory mediators
8	KE	459	Liver Steatosis	Increased, Liver Steatosis
9	KE	1954	Gut microbiota alteration	Gut dysbiosis
10	KE	1549	Liver injury	Liver injury
11	KE	1997	Colonic epithelial lesions	Colon epithelial lesions
12	AO	1996	Increased morbidity and mortality of digestive disease	Increased morbidity and mortality of digestive disease

*MIE* molecular initiating events, *KE* key events, *AO* adverse outcomes

**Table 4:** Summary of the AOP of neurologic system

Sequence	Type	Event ID	Title	Short name
1	MIE	1115	ROS generation	Increased, Reactive oxygen species
2	MIE	151	Inflammatory pathways Activation	Inflammatory pathways Activation
3	KE	1392	Oxidative stress	Oxidative stress
4	KE	1239	Altered gene expression	Altered, Gene Expression
5	KE	191	Neuronal dysfunction	Neuronal dysfunction
6	KE	149	Inflammatory response	Increase, Inflammation
7	KE	1496	Secretion of inflammatory factors	Increased proinflammatory mediators
8	KE	1998	Microglia activation	Microglia activation
9	KE	1514	Neurodegeneration	Neurodegeneration
10	KE	188	Neuroinflammation	Neuroinflammation
11	AO	1999	Neurodegenerative disease	Neurodegenerative disease
12	AO	402	Decreased cognitive Function	Cognitive Function, Decreased

*MIE* molecular initiating events, *KE* key events, *AO* adverse outcomes

**Table 5:** Summary of the AOP of reproductive system

Sequence	Type	Event ID	Title	Short name
1	MIE	1115	ROS generation	Increased, Reactive oxygen species
2	MIE	151	Inflammatory pathways Activation	Inflammatory pathways Activation
3	KE	1392	Oxidative stress	Oxidative stress
4	KE	1815	Activation of ER stress	ER stress
5	KE	1945	Dysfunctional autophagy	Dysfunctional Autophagy
6	KE	177	Mitochondrial dysfunction	Mitochondrial dysfunction
7	KE	149	Inflammatory response	Increase, Inflammation
8	KE	1496	Secretion of inflammatory factors	Increased proinflammatory mediators
9	KE	1758	Impaired Spermatogenesis	Impaired, Spermatogenesis
10	KE	1837	Decreased oocyte maturation	Decreased oocyte maturation
11	AO	520	Decreased sperm quantity or quality	Decreased sperm quantity or quality
12	AO	363	Altered reproductive behaviour	Altered, Reproductive behaviour

*MIE* molecular initiating events, *KE* key events, *AO* adverse outcomes

Combined with the epidemiological and laboratory evidence, this review comprehensively illustrated the potential molecular mechanisms of health injury caused by PM and its components in various systems, as well as the combined toxicity of PM with other air pollutants. The main conclusions and new insights on the correlation between public health and PM were summarized, especially at low concentration exposure, which may provide enlightenment for the treatment and prevention of environmental-related diseases, contribute to the development of clinically relevant drugs and provide certain guidelines for future research directions.

### The effects and mechanisms of PM on respiratory system

As the initial site of deposition, the respiratory system is the primary target of PM_2.5_. Increasing evidence suggests that PM_2.5_ can cause damage to the respiratory system, leading to lung function impairment and inducing the occurrence and development of chronic obstructive pulmonary disease (COPD), asthma, pulmonary fibrosis and other respiratory diseases [43–45]. Epidemiological studies have shown that exposure to PM_2.5_ can cause pneumonia (KE 1993), lung cancer, increased morbidity and mortality of respiratory diseases (KE 1992) and decreased lung function (KE 1250) [46–49]. Therefore, it is crucial to deeply understand the current status of PM_2.5_ effects on the respiratory system and its mechanisms.

### Metabolic activation

PM_2.5_ could enter the cells [50, 51]. The organic chemicals (such as volatile organic compounds) of PM_2.5_ could activate the aryl hydrocarbon receptor (AHR) (KE 18) in the target cells, which results in apoptosis (KE 1262) through DNA damage (KE 1194) and the overexpression of AHR regulatory genes (KE 165) such as cytochrome P450 enzymes (CYP1A1, CYP1A2, CYP1B1, CYP2E1, CYP2F1) [52–54]. Subsequently, the organic chemicals of PM_2.5_ could be metabolized and activated to the reactive electrophilic metabolites (REMS) by these xenobiotic metabolic enzyme systems, which will produce various toxic effects on target cells [54, 55].

### Inflammatory response (KE 149)

The inflammatory response was found to participate in the process of respiratory injury caused by PM_2.5_. Studies have confirmed that PM_2.5_ could induce pulmonary inflammatory injury by infiltration of inflammatory cells (KE 1497) and release of inflammatory factors [34, 56]. In addition, short-term exposure to PM_2.5_ may induce epithelial-mesenchymal transition (KE 1650) through inflammatory pathways activation (KE 151), mediating the development of pulmonary fibrosis (KE 1458) [57]. Alveolar macrophages and pulmonary epithelial cells are important target cells of PM_2.5_. After being phagocytized and wrapped by alveolar macrophages, multiple components of PM_2.5_ could enhance the activity of alveolar macrophages (KE 1198) and induce the secretion of tumor necrosis factor-α (TNF-α), interleukin-6 (IL-6) and interleukin-8 (IL-8), causing inflammatory response [58]. The water-soluble substances and organic components of PM_2.5_ could induce the release of IL-6 in A549 and BEAS-2B cells [59]. In addition, the metal components of PM_2.5_ could cause abnormal expression of miRNA in the alveolar macrophages and bronchial epithelial cells, indirectly resulting in lung injury by regulating the expression of inflammatory genes (KE 151), leading to respiratory damage [60, 61].

### Oxidative stress (KE 1392)

Under normal conditions, tissues and organs were in a state of oxidation-antioxidant balance. Numerous oxygen free radicals can be generated by external harmful stimuli, resulting in an imbalance of the redox state, which can directly or indirectly induce respiratory diseases. It was found that organic chemicals on the surface of PM_2.5_ were metabolically activated into REM, which may lead to the increase of intracellular ROS (KE 1115) [54]. Heavy metals and PAH of PM_2.5_ could stimulate airway epithelial cells to produce amounts of ROS, leading to oxidative damage to the tissues [62, 63]. In addition, studies have also proposed that the metal components of PM_2.5_ could induce the generation of hydroxyl free radicals, which cause the peroxidation of unsaturated fatty acids on the cell membrane and reduce the fluidity of the cell membrane, leading to altered membrane permeability and cell membrane damage [64]. One study revealed that the ROS production induced by PM was reduced by 36% after removing the metal component of it [65].

### Immune injury

The immune system is the first line of defense against external pathogenic factors. A recent study suggested that prolonged exposure to PM_2.5_ could reduce influenza virus resistance (KE 1738) by downregulating the histone demethylase Kdm6a of pulmonary macrophages and mediating histone modification in the IL-6 promoter region [66]. Animal studies have shown that long-term exposure to PM_2.5_ could inhibit the production of inflammatory cytokines induced by pneumococci, which increases the risk of pneumococcal disease [67]. Moreover, a study revealed that the gene expression (KE 1239) of C-reactive protein (CRP), IgM, IgG and IgE was enhanced, as well as the IgA and CD8^+^ expression was decreased in the population serum due to long-term exposure to automobile exhaust PM_2.5_, suggesting that PM_2.5_ can cause inflammatory response and immune injury [68].

### DNA damage (KE 1194)

PM_2.5_ can cause the occurrence and development of respiratory diseases by DNA damage [69]. Exposure to a high concentration of PM_2.5_ could affect the activation of multiple gene pathways in human bronchial epithelial cells, which are related to inflammation and immunity. Besides, most of the down-regulated genes are related to the functions of defense, phagocytosis and repair [70, 71]. Some studies have shown that PM_2.5_ can induce amounts of ROS production in cells, which leads to intracellular DNA fusion, DNA double-strand break, mitochondrial and spindle damage, resulting in mitotic arrest, cell death and changes in genetic information [52]. Other studies revealed that the metabolites of PAH could covalently bind to the amino terminus of the guanine outer ring of DNA nucleophilic sites to form adducts, causing DNA damage, gene mutations and cell carcinogenesis [72]. Finally, one study suggested that metal dust of PM_2.5_ could cause cell deformation, inhibition of cell proliferation and DNA damage, which would then cause cell cycle arrest or cell apoptosis [73].

### Apoptosis (KE 1262)

Apoptosis plays an important role in the growth and development of organs and tissues, immunity, metabolism as well as the clearance of abnormal cells. However, the disorder of the apoptosis process may be directly and indirectly related to various diseases. It was reported that PM_2.5_ was able to induce apoptosis of alveolar epithelial cells and subsequently cause pulmonary fibrosis [74]. One study showed that the apoptosis rate of human bronchial epithelial cells increased from 3.8 to 66.7% after exposure to PM_2.5_ for 24 h. Moreover, this study confirmed that the components of PM_2.5_ (heavy metals, microorganisms, etc.) also played a key role in the process of inducing apoptosis [75]. In addition, PM_2.5_ from cooking oil fume could induce excessive apoptosis of alveolar type II epithelial cells through the ER pathway [76]. Other studies have comprehensively explored the cytotoxicity of A549 cells induced by PM_2.5_ at the proteomic level. The results showed that oxidative stress, metabolic disorder, signal transduction disorder, abnormal protein synthesis or degradation and cytoskeleton disorder were the main factors of A549 cytotoxicity induced by PM_2.5_ and further suggested that PM_2.5_ could induce apoptosis of A549 cells through p53, c-Myc and p21 pathways [77].

### Autophagy (KE 1945)

Autophagy is a physiological or pathological process in which cells encapsulate damaged organelles and proteins in a specific membrane structure and then send them into lysosomes for degradation [78]. Long-term PM_2.5_ exposure can lead to lung inflammation and pulmonary fibrosis, of which the molecular mechanism may be related to dysfunctional autophagy [79]. One study suggested that exposure to PM_2.5_ specifically increased the expression of nitric oxide synthase 2 (NOS_2_) and the production of NO in human bronchial epithelial cells, which led to excessive autophagy in cells, and that blocking NOS_2_ could effectively inhibit the excessive autophagy and cell death [80]. Autophagy was also observed in A549 cells cultured in vitro after PM_2.5_ treatment. In addition, with the elevation of the concentration and time of PM_2.5_ exposure, the expression of the autophagy-related protein LC3 was up-regulated [81]. After exposure to the organic extracts of PM_2.5–0.3_ for 24 h, the expression of ATG5 and Beclin1 was decreased in BEAS-2B cells, which are essential for the autophagy process [82]. This result indicated that the organic extract of PM_2.5–0.3_ affected autophagy.

Currently, mounting studies are being performed on circRNAs, which are considered to be competing endogenous RNAs (ceRNAs) that bind to miRNAs to affect gene expression through complementary base pairing. After exposure to PM_2.5_, circBbs9 was upregulated and bound to miR-30e-5p, thereby elevating the expression of inflammation cytokines, resulting in pulmonary inflammation through the activation of the NLRP3 inflammasome [83]. In addition, a recent study revealed that PM_2.5_ can cause lung injury by inducing suppression of ferroptosis (KE 1994) and lipid peroxidation (KE 1445). The KeaP1-Nrf2-SLC7A11/GPX4 pathways were involved in this process [84].

All in all, there has been much research on the mechanisms of the occurrence and development of respiratory diseases induced by PM_2.5_, but further improvement is needed. Emerging mechanisms still need to be discovered in the research on the pathogenic process of PM_2.5_. Further investigations of the mechanisms of the effects of exposure to low-concentration PM on the respiratory system are crucial. Early effect markers of PM on respiratory system also need to be explored for early risk assessment. Moreover, we should make full use of these conclusions to further explore methods and technologies to effectively prevent and treat the harmful effects on respiratory system caused by PM_2.5_, improving the health level of residents.

### The effects and mechanisms of PM on cardiovascular system

PM_2.5_ deposited in alveoli can directly enter the circulatory system through the blood-air barrier, which is harmful to cardiovascular health, and eventually causes serious damage to blood vessels and heart. Epidemiological and clinical studies have shown the association between PM with increased morbidity and mortality of cardiovascular diseases (CVDs). Exposure to PM can induce systemic oxidative stress, inflammation, vascular dysfunction, thrombosis and coagulation, leading to a series of CVDs (KE 1929), such as myocardial obstruction, atherosclerosis, heart failure, thrombosis, ischemic heart disease and coronary artery disease [85–89].

### Toxicity of PM to the cardiovascular system

PM_2.5_ can cause cardiovascular damage through a variety of mechanisms, such as oxidative stress, inflammatory response, apoptosis, autophagy, epigenetic modifications, etc. Studies have shown that PM_2.5_ can cause the destruction of the balance of oxidants and antioxidants by up-regulating the production of intracellular ROS (KE 1115) and the expression of oxidative stress-related genes, leading to oxidative stress (KE 1392), including the reduction of superoxide dismutase activity, the release of lactate dehydrogenase and the increase of cell membrane permeability. Subsequently, activation of related pathways can cause cardiac fibrosis (KE 1924), significantly enhance vascular endothelial permeability and other cardiovascular damage, leading to CVD [90–93]. Inflammatory pathways activation (KE 151) and systemic inflammation (KE 149) were observed after exposure to PM_2.5_, which in turn led to increased secretion of a range of pro-inflammatory factors (KE 1496) and vascular endothelial dysfunction (KE 1928) [94, 95]. PM_2.5_ significantly decreased the expression of vascular endothelial growth factor receptor 2 (VEGFR2) as well as elevated the expression of somatostatin (SST) and its receptor, inducing endothelial inflammation and significantly inhibiting the migration and cell viability of endothelial cells. Additionally, PM_2.5_ can reduce the repair ability of vascular endothelial cells by inhibiting endothelial cell proliferation and migration [96]. Apoptosis is an important mechanism of cardiovascular toxicity induced by PM_2.5_. Studies have shown that PM_2.5_ increased the apoptosis level of human aortic endothelial cells (HAEC) by decreasing the Bcl-2/Bax ratio and increasing the expression of cytochrome C, caspase-9 and caspase-3 [97]. PM_2.5_ can negatively regulate the IRAK2/TRAF6/NF-κB signaling pathway through miR-205 to induce apoptosis (KE 1918) of cardiomyocytes (AC16), resulting in interstitial edema and destruction of myocardial fiber [98]. Besides, oxidative stress and calcium overload induced by PM_2.5_ are the causes of endoplasmic reticulum (ER) stress and mitochondrial dysfunction (KE 177), which further lead to endothelial cell apoptosis and cardiovascular toxicity [99]. PM_2.5_ was found to induce the blockage of autophagic flux (failure of fusion between lysosomes and autophagosomes), which is detrimental to the survival of endothelial cells. Moreover, PM_2.5_ was able to disrupt the normal pathway of autolysosome formation, causing autophagy defects and the dysfunction of autophagy (KE 1927) flux, which can aggravate endothelial cell damage [100]. Ning et al. showed that PM_2.5_ increased mitochondrial oxidative stress and activated mitophagy, leading to mitochondrial dysfunction and mitochondrial dynamics disorder, which resulted in the synthetic phenotype remodeling of vascular smooth muscle cells (VSMCs) and ultimately aortic fibrosis [101]. In addition, PM_2.5_ can induce differential methylation of genes related to cardiovascular development, vascular size regulation, vascular development and other pathways, which are associated with heart-related diseases [102]. Yang et al. showed that PM_2.5_ can affect the expression (KE 1239) of myocardial ADRB2 by inducing hypermethylation, which in turn activates β2AR/PI3K/Akt pathway, leading to myocardial apoptosis and cardiac dysfunction (KE 1919) [103].

In recent years, some emerging mechanisms have been progressively noticed to play a role in the cardiovascular toxicity of PM. Ferroptosis (KE 1994), a newly proposed form of cell death, mainly depends on the signaling of lipid peroxidation (KE 1445) and intracellular iron overload. A recent study demonstrated that PM_2.5_ increased iron content and production of ROS in endothelial cells, leading to intracellular lipid peroxidation, iron overload and redox imbalance, followed by ferroptosis and secretion of inflammatory cytokines [37]. Additionally, PM_2.5_ could lead to down-regulation of lncRNA (PEAMIR) expression in heart tissue, of which the effect as competing endogenous RNAs (ceRNAs) was reduced, and thus weakened the inhibition of miR-29b-3p, leading to elevation of cardiac inflammation and apoptosis [104]. An increased level of acetylated histone 3 lysine 9 (H3K9ac) was also found to be involved in the cardiovascular toxicity of PM_2.5_, resulting in myocardial injury and upregulation of inflammatory factors [105].

### Cardiovascular toxicity mechanisms of the PM components

It is well known that the toxicity of PM may depend on its chemical composition [106]. Wu’s study has shown that certain PM_2.5_ metals and chemicals are more closely associated with circulating biomarkers of endothelial dysfunction compared with PM_2.5_ [107]. The components of PM_2.5_ such as several metals (Fe, Pb, Ni and Zn) and elemental carbon (EC) were positively correlated with ischemic heart disease (IHD) [108]. A multicenter study of 11 European cohorts noted that transition metal components in PM_2.5_ such as Fe, Ni, V and their potential to produce ROS may contribute significantly to the burden of oxidative stress. Furthermore, the elevated risk of coronary events was observed to be associated with chronic exposure to PM_2.5_ components, especially K, Si and Fe [109]. Besides, long-term exposure to Fe and Cu in PM_2.5_ as well as their combined effects on ROS were consistently associated with increased death of CVD [110]. Zhang et al. demonstrated in vitro that heavy metals bound to PM_2.5_ induced apoptosis of rat H9C2 cells through ROS-mediated inflammatory response, resulting in cardiotoxicity [111]. Long-term exposure to transition metals in PM is associated with elevated concentrations of inflammatory blood markers such as high-sensitivity C-reactive protein (hsCRP) and high fibrinogen levels, which can lead to chronic systemic inflammation and increase the risk of CVD [112]. Further studies have found that Ni, the transition metal component in PM_2.5_, synergistically increases systemic and vascular oxidative stress, which can exacerbate PM_2.5_ exposure-related endothelial dysfunction [113, 114].

### Joint exposure mechanisms of PM and other environmental pollutants

Due to the complexity of environmental air pollution components, the study of the combined toxicity of PM_2.5_ and other environmental pollutants is of great importance. Zhang et al., indicated that co-exposure to SO_2_, nitrogen dioxide (NO_2_) and PM_2.5_ could induce endothelial dysfunction by increasing inflammatory response, resulting in decreased blood pressure and increased heart rate in mice [115]. Besides, exposure to both ozone (O_3_) and PM_2.5_ can lead to changes in autonomic nerve balance, which in turn increase arrhythmias and mechanical decrements, resulting in impaired heart function in mice [116, 117]. In addition, exposure to PM_2.5_ and acrolein together caused myocardial dyssynchrony in mice by activating transient receptor potential cation channel A1 (TRPA1), which adversely affected cardiac function and increased the risk of CVD [118].

In brief, oxidative stress, inflammation, apoptosis and autophagy are the main mechanisms of cardiovascular injury caused by PM. Besides, emerging evidence suggests that ferroptosis and epigenetic modification also participate in the adverse effects of PM. In addition, the metal component of PM can exacerbate the toxicity of it. In recent years, some epidemiological evidence has confirmed that low-concentration PM exposure is still positively associated with CVD. This suggests that further exploration should be carried out to reveal more potential mechanisms and provide more sufficient evidence for cardiovascular injury caused by low-concentration exposure to PM based on existing evidence.

### The effects and mechanisms of PM on digestive system

In recent years, studies have shown that the digestive system can be directly or indirectly exposed to PM. After inhalation, larger particles of PM were isolated in the upper or conductive lower respiratory tract, while smaller particles, especially PM_2.5_, could be phagocytosed by macrophages in the phagocytosed and alveolar spaces. The particles isolated by macrophages and adsorbed in the mucus layer of the lower respiratory tract were then transported back to the oropharynx and eventually swallowed into the gastrointestinal tract. Another route for PM to enter the gastrointestinal tract is the direct dietary intake of food and water contaminated by PM pollution [119, 120]. Epidemiological evidence has also suggested that PM is a risk factor for hospital outpatient visits for digestive diseases, and there is a significant association between PM_2.5_ and the mortality of digestive diseases (KE 1996) such as liver steatosis (KE 459), stomach and colorectal cancer [121, 122].

### Toxicity mechanisms of digestive tract

After entering the gastrointestinal tract, PM can change the morphology and function of the gastrointestinal epithelium as well as the composition of gut microbiota, thus injuring the digestive tract system [123, 124]. Therefore, the impact of PM on intestinal health is attracting more and more attention. Current studies on animal particulate exposure have identified mechanisms involving homeostasis components in the gut. Colonic epithelial lesions (KE 1997) were observed in mice, which are continuously exposed to urban PM. Meanwhile, the upregulation of key molecules of the inflammatory pathway (KE 151) (Stat3 and P65) and colonic infiltration of inflammatory cells (KE 1497) were also observed under these conditions [125, 126]. Studies have shown that the antioxidant effects of D-4F peptide (a mimic of apolipoprotein A-I) and N-acetyl-L-cysteine can mitigate PM-mediated intestinal injury with the involvement of an unbalanced intestinal redox pathway [127, 128]. Harmful effects of PM inhalation on the gut microbiota (KE 1954) have also been reported. Using a multifunctional aerosol concentration enrichment system, a meta-genomic analysis of the fecal microbiota of mice exposed to high PM_2.5_ concentrations in Shanghai, China, for 12 months revealed differences in the abundance of 24 bacteria and 21 fungi compared with control animals, suggesting that long-term exposure to PM_2.5_ could lead to intestinal dysbiosis [128]. PM exposure alters the microbial composition of the entire gastrointestinal tract, with a more pronounced ecological imbalance from proximal to distal, which in turn triggers mucus depletion and subsequent colon epithelial damage, as well as inflammatory infiltration [126]. Studies have also suggested that PM leads to changes in the intestinal structure: villi length decreased in LDL-deficient mice exposed to UFP; Apolipoprotein E deficient (ApoE)^−/−^ mice on a high-fat diet were exposed to wood smoke or mixed diesel and gasoline vehicle exhaust with reduced Muc2 and tight junction protein expression [129]. Similarly, in mouse models of Alzheimer's disease, PM_2.5_ exposure aggravated intestinal histopathological damage and stimulated the secretion of pro-inflammatory cytokines [130]. Mechanistically, FGFR4-triggered activation of the PI3K/AKT pathway plays a key role in PM acceleration of colorectal tumor formation. At the same time, a glycoprotein called carbonic anhydrase 9, involved in colorectal cancer development, was upregulated in the colon of mice exposed to DEP, suggesting adverse health effects on the digestive tract [131].

### Toxicity mechanisms of digestive glands

Epidemiological studies have shown that long-term exposure to PM_2.5_ was associated with elevated levels of liver enzymes, particularly ALT and GGT, which suggested that PM_2.5_ has potentially adverse effects on liver function [132]. Through in vitro and in vivo experiments, Xu et al. found that inflammation (KE 149) caused by long-term PM_2.5_ exposure was involved in dyslipidemia-related chronic liver injury (KE 1549), and the levels of IL-1β, IL-6, IL-18 and TNF-α in liver tissue were significantly increased (KE 1496) [30]. In addition, insoluble particles of PM_2.5_ can cause abnormal liver function by inducing inflammatory signal transduction and an increase of cytokines in liver, accompanied by infiltration of inflammatory cells and macrophages. In addition, exposure to PM_2.5_ can induce up-regulation of cell proliferation with the increase of hepatocyte proliferation markers, hepatocyte balloon degeneration and nuclear enlargement signals [133]. A microfluid-controlled liver and kidney microphysiological system (LK-MPS) was used to confirm that PM_2.5_ disrupted classic IRS-1/AKT signaling pathways (INSR, PI3K, AKT, IRS-1, GLUT2, GLUT4 and FOXO1 downregulation) as well as IR-related metabolic pathways: lipid biosynthesis (ceramide and triacylglycerol), gluconeogenesis (β-d-glucose 6-phosphate) and lipid biosynthesis (ceramide and triacylglycerol) pathways, leading to dysregulation of glucose levels and aggravate hepatic insulin resistance [134]. Consistently, exposure to PM_2.5_ resulted in increased expression of Bmal1, Cry1 and Reverbα in the liver. In addition, the enhanced expression of PPARα in mouse liver induced by PM_2.5_ leads to up-regulated fatty acid transport and oxidative stress (KE 1392). Finally, the expression of lipid-synthesis rate-limiting enzymes in the livers of PM_2.5_-exposed mice was significantly increased, suggesting the occurrence of abnormal lipid metabolism (KE 1995) [135].

In general, the adverse effects of PM on the digestive system are mainly caused by inflammation, redox imbalance and abnormal lipid metabolism, which result in liver injury as well as changes of gastrointestinal epithelial morphology and the composition of intestinal microbes. However, it can be seen that the current research on the effects of PM on digestive system is still at a relatively superficial level. The interference of PM on deeper mechanisms such as cell death patterns, metabolic pathways and other functions, which are related to digestive system, has not been fully investigated.

## The effects and mechanisms of PM on neurologic system

### Toxicity of PM to the neurological system

In recent years, a growing number of epidemiological studies have reported the association between air pollution exposure and neurodevelopmental disorders and neurodegenerative disease (KE 1999), including slow cognitive development, attention deficit hyperactivity disorder (ADHD) and autism spectrum disorder (ASD) in children, as well as cognitive decline and schizophrenia in adults [136, 137]. PM, as one of the most important air pollutants, may accelerate brain aging and increase the risk of dementia [138]. Numerous epidemiological studies have identified the adverse effects of PM on cognitive function (KE 402), which can negatively affect the central nervous system, causing neurological or psychiatric disorders. A cohort study confirmed that exposure to PM during pregnancy resulted in psychomotor retardation in the offspring as they grew up [139]. Moreover, studies have shown that the levels of neuroinflammatory biomarkers are significantly increased in the brains of children with cognitive deficits after exposure to high levels of PM [140]. In addition, population studies in Pennsylvania, Ohio, Taiwan, Shanghai, China, and Denmark have all reported that PM_2.5_ can increase the risk of Alzheimer's disease [141–145].

Animal studies have suggested that inhalation of PM_2.5_ can cause various neurological disorders associated with the basis of episodic memory processes, such as Alzheimer's disease and Parkinson's syndrome [146, 147]. The reason may be that ambient PM pollution accelerates amyloid β accumulation and neurofibrillary tangles [148]. But the underlying mechanism remains unclear [149]. Mice living in highway tunnels, which can breathe more traffic-related PM, had higher levels of pro-inflammatory cytokines (KE 1496) in their brains [150]. Elevated α-synuclein may be a mechanism by which risk factors such as aging increase the vulnerability to neurodegeneration (KE 1514). With transgenic mice overexpressing α-synaptic nuclear proteins, a marker of Parkinson's disease, it has been observed that PM increases the expression of synaptic nuclear proteins [151]. There is also strong evidence for the association between PM and Alzheimer's disease. Persistent exposure of pregnant mice to PM for 25 weeks resulted in microglial activation (KE 1998), impaired hippocampus, altered structure and function of the blood–brain barrier in brain tissue, as well as increased behavior and situational memory deficits in offspring male rats [152]. In addition, studies have shown that prenatal exposure to PM_2.5_ can alter the structure of the cerebral cortex, normal behavior and hormone levels in the brains of offspring mice [153, 154]. Rats exposed to PM_2.5_ after birth also develop neurological symptoms, such as communication disorders, poor social interaction and avoidance of new things, while activating microglia and increasing the secretion of pro-inflammatory cytokines [155]. Although the exact mechanism by which PM causes neurotoxicity is still elusive, it is currently clear that PM can cause microglia activation, oxidative stress (KE 1392) and neuroinflammation (KE 188). PM_2.5_ increased c-Jun N-terminal kinase (JNK) phosphorylation (KE 151) and decreased Akt phosphorylation and re-formed microglia through increasing neuroinflammation, proinflammatory M1 expression and disease-associated microglia phenotypes, leading to neurotoxicity [156]. PM_2.5_ can increase the specific DNA hydroxymethylation and overall DNA hydroxymethylation of neurons, which subsequently interferes with their mRNA expression (KE 1239), further leading to reduced length of the neurites and neuronal dysfunction (KE 191). The results after blocking with antioxidants indicate that oxidative stress-mediated hydroxymethylation is involved in PM_2.5_-induced axon growth and synapse formation defects [5]. Similarly, genetic analysis of human monocytes reported that exposure to PM can cause neurotoxicity [157].

### ***Neurological toxicity mechanisms of the PM***_***2.5***_*** components***

PM is one of the most dangerous pollutants, not only because of its aerodynamic diameter but also because it contains many toxic components. The smaller the aerodynamic diameter of PM is, the easier it is to enter the body and cause damage. At the same time, a smaller diameter leads to a larger surface area that can adsorb more toxic components [158]. Population studies have shown that the exposure level of manganese (Mn) carried by PM_2.5_ in ambient air is significantly lower than current occupational exposure thresholds in the United States, but it is still correlated with neurotoxicity and brain injury, which are primarily manifested in Parkinson's syndrome [159]. A cross-sectional study in Brazil revealed that Pb-Mn co-exposure can cause mental retardation in children living in industrial areas [160]. Vanadium, as a transition metal, is mainly emitted into the atmosphere through the combustion of fossil fuels and then enters the alveolar sacs to produce toxicity through adsorption on the surface of PM [161]. Studies have shown that vanadium can induce oxidative stress in the central nervous system and lead to structural and functional changes, such as apoptosis of neurons in olfactory bulb and decrease of dendritic spines in granulosa cells [162, 163], as well as cause the loss of ependymal epithelial cilia in the ventricle and the remaining cilia are compactified, leading to the desquamation of cells at the junction and eventually the general collapse of tissue structure [164]. These studies suggest that vanadium can impair the blood–brain barrier, making the nervous system more vulnerable to injury and further increasing the risk of PM and other components of it. Increases in serum glutathione and neuronal cell death in the nucleus accumbens have been observed in the brains of men exposed to UFPs. Similarly, Fe and S levels have been increased in the brains of male mice exposed to Fe and S-rich UFPs, leading to ferroptosis and oxidative stress [165]. Compared with eluted PM, total PM contains more PAH and metal content, which can cause increased NF-κB expression and nuclear localization of it in the brain of male mice, inducing more brain inflammation and depression [166]. Studies have found that PM_2.5_ and its extracts, including carbon core components, organic extracts and water-soluble extracts, could cause cell proliferation inhibition, cell apoptosis and cell cycle arrest in neuronal cells in varying degrees. This indicates that the toxic compounds adsorbed on the particles may induce various types of brain injury through oxidative damage.

PM_2.5_ and the organic extracts of it can interfere with mRNA expression by increasing specific DNA hydroxymethylation and overall DNA hydroxymethylation of neuronal genes, which subsequently cause neuronal developmental disorders including reduced neurite length, axon outgrowth and defective synapse formation. The neurocytotoxicity of different components still requires further assessment [5]. There is increasing evidence to suggest that PM and its components are associated with memory disorders, communication disorders and anxiety/depression, either from co-exposure to PM and heavy metals or from combined exposure to heavy metals. The combined effect of these toxic ingredients increases the risk of neurodegenerative diseases, ischemia and cognitive decline [160, 167, 168].

### ***Joint exposure mechanisms of PM***_***2.5***_*** and other environmental pollutants***

Not only PM, but other components of air pollution, including toxic gases as well as organic and inorganic compounds, can also cause neurotoxicity [169]. Especially, various pollutants will react with each other to form joint exposure mechanisms. As a potential cause of neurodegenerative (such as Alzheimer’s disease) and neurodevelopmental (such as autism spectrum disorders) disorders, air pollution may cause damage through oxidative stress and neuroinflammation [170, 171]. The most significant damage they cause to humans and animals is the activation of microglia, increased lipid peroxidation and neuroinflammation in the brain, especially in the hippocampus and olfactory bulb [172–174]. Cross-sectional studies have also shown that PM_2.5_ is correlated with O_3_ and NO_2_, causing cognitive impairment in middle-aged and elderly people in Los Angeles [175]. Exposure to O_3_, PM and their mixtures have been demonstrated to have neurotoxic effects in various animal models [176, 177]. Neurotoxicity of air pollution mixtures accelerating brain aging has been found in experimental dogs raised in Mexico City, which mainly affects the olfactory and nasal mucosa of respiratory tract, olfactory bulbs and cerebral cortex. Air pollution was characterized by mixtures of O_3_, aldehydes, PM and other components [178]. Exposure to PM_2.5_ alone can lead to inflammation and endothelial function impairment in rats, while combined exposure with O_3_ will enhance these effects [179]. The combined exposure to PM and Staphylococcus aureus will lead to abnormal metabolism of cholinergic system enzymes and neurotransmitters as well as elevated expression of pro-inflammatory cytokines and neurotrophins in mice, which will further cause impaired motor function, impaired learning/memory abilities and other neurotoxicity [180]. In rodent midbrain neuron-glial cell cultures, UFPs and rotenone can cause neuronal death in a dose-dependent manner, respectively. However, when the non-toxic doses of UFPs and rotenone are jointly exposed, they will synergistically activate the NADPH oxidase in microglia, resulting in oxidative damage to dopamine neurons [181].

In short, exposure to PM increases the risk of neurodevelopmental disorders, neurodegenerative diseases and cognitive decline by inducing activation of microglia, lipid peroxidation, neuroinflammation and neuronal death. The mechanisms may involve inflammation, oxidative stress, apoptosis and epigenetic modification. In addition, recent evidence suggests that ferroptosis is an emerging mechanism for the neurotoxicity of PM. At present, there is some evidence about the mechanism of nervous system injury caused by PM, and new mechanisms are being discovered. However, the potential association between PM and the adverse effects on nervous system, including accelerated amyloid β accumulation and neurofibrillary tangles, remains to be explored.

### The effects and mechanisms of PM on immune system

Ambient air pollution consists of PM and gaseous components. The former includes nitrogen oxides (Nox), carbon monoxide (CO), volatile organic compounds (VOCs) and O_3_. Major constituents of PM are mineral dust, organic and elemental/black carbon, as well as inorganic ions such as NO_3_^−^, SO_4_^2−^ and NH_4_^+^. Other minor constituents, such as heavy metals, can be found in PM at low concentrations [182]. In recent decades, PM pollution has become increasingly serious and prominent, causing a variety of threats to human health and being one of the most serious hazards to human health. The immune system is considered to be the most vulnerable target of air pollutants, which participate in many pathological conditions. Increasing data suggest that PM_2.5_ is associated with immunotoxicity, which can cause some serious damage [183].

### ***Studies on immunotoxicity caused by PM***_***2.5***_

Exposure to PM can cause immunotoxicity. A study illustrated that PM_2.5_ triggered ER stress and oxidative stress in the spleen of SD rats and resulted in apoptosis through up-regulating CHOP and caspase-12 signaling pathways. Specifically, PM_2.5_ significantly increased LC3 expression and decreased p62 expression, which activated autophagy in the spleen of SD rats in a concentration-dependent manner and finally induced immunotoxicity [183]. A significant inflammatory response was observed 5 days after exposure to PM_2.5_. Monocytes/macrophages in lung tissue and bronchoalveolar lavage fluid showed a transient response, while neutrophils showed a cumulative response. Additionally, exposure to PM_2.5_ led to increased expression of monocyte chemoattractant protein 1 (MCP-1) cytokine, which is an attractant of monocyte/macrophage in blood. These findings demonstrated that PM_2.5_ can induce inflammatory responses in which macrophages and neutrophils are involved [184]. Other studies have suggested that parental PM_2.5_ could mediate Th17- and T regular-related immune microenvironment changes, leading to increased blood pressure in offspring [185]. In vitro, studies have shown that PM_2.5_ may exacerbate viral myocarditis through MMP-2/TIMP-1 imbalance, perforin response and Th17-mediated viral replication [186]. Exposure of PM_2.5_ to Jurkat T cells mediated a local increase of Ca^2+^ production by causing elevated expression of Orai1 and CaN-NFAT genes, the cytoplasmic concentrations of TNF-α and IL-2 may also be changed [187]. Th1/Th2 balance plays an important role in lung and systemic inflammation associated with air pollution. Considering the mechanism of immune system toxicity caused by PM_2.5_, Hou et al. found that the imbalance of T lymphocyte immunity caused the increase of related microRNA profiles, driving a Th1-biased immune response after acute PM_2.5_ exposure [188]. PM_2.5_ activated the NF-κB signaling pathway in A549 as well as HEK293 cells and increased the expression of Nod1. The results indicated that PM_2.5_ might contribute to chronic allergic diseases by stimulating the innate immune system through the PM_2.5_-Nod1-NF-κB axis [189]. GFAP, which is a sign of astrocyte activation, has enhanced immunoreactivity and the content of cytokines in the spleen also changes with the increase of PM_2.5_. These findings suggested that PM_2.5_ might influence the development of the immune system [190]. Research has shown that PM_2.5_ causes cytotoxicity of airway epithelial cells as well as activates bone marrow-derived antigen-presenting cells (APCs) and T-cells in mice, which finally leads to the deterioration of respiratory diseases [191]. It is revealed that the levels of TNF-α, IL-6 and IL-8 in A549 cells increased significantly after PM_2.5_ treatment. Further studies showed that the TLR4-NF-κB p65 signaling pathway was involved in the inflammatory reaction induced by PM_2.5_. Besides, PM_2.5_-induced autophagy promoted the inflammatory response by increasing NF-κB p65 level. And autophagy deficiency enhanced the expression of Nrf2, which can inhibit the inflammatory response as well as the levels of IL-8, IL-6 and TNF-α induced by PM_2.5_ stimulation [192]. In CpG-DNA (TLR9 ligand)-stimulated dendritic cells, PM_2.5_ significantly reduced the levels of TNF-α, IL-6 and IL-12 p40, which might be related to the inhibition of NF-κB and MAPK signaling pathways [193]. It has been suggested that PM_2.5_ and formaldehyde might induce hematopoietic toxicity by reducing the expression of blood cells, myeloid progenitor cells (MPCs) and hematopoietic growth factors. In addition, PM_2.5_ increased DNA damage and oxidative stress by disrupting the balance of Treg/Th17 and Th1/Th2 and suppressing the DNA-repair related mTOR pathway [194].

### The immunotoxicity caused by PM components and other pollutants

In recent years, it has been suggested that the toxicity of chemical components carried by PM may be more closely related to its toxic effect. Regarding the toxicity of PM components, it has been reported that not only the pure particulate components of PM_2.5_ (mainly composed of carbon) but also the ambient particulate components (original PM_2.5_ particles) can cause acute toxicity, especially inflammatory responses, which involve the accumulation of immune cells as well as the elevation of the levels of pro-inflammatory cytokines in lungs. Furthermore, original PM_2.5_ had a stronger ability to induce inflammatory response than pure particulate fractions [195]. Moreover, a study of PM components found that exposure to urban PM from both Baltimore and New York City could stimulate mixed Th2/Th17 inflammation in the airways of mice. More potent airway inflammation was triggered by PM, accompanied by cytokine release (IL-5, IL-17A, IFN-γ and IL-13), inflammatory infiltrate and hyperresponsiveness, which may reflect a higher metal content, while particulate components such as coal fly ash, DEP and carbon black were not capable of the above inflammatory effects [196]. Due to its greater PAH content and higher oxidant potential, UFPs are more likely than PM_2.5_ to enhance the ability of secondary ovalbumin (OVA) to induce allergic airway inflammation, leading to mucoid hyperplasia and eosinophilic inflammation from nasal turbinates to small pulmonary airways [197]. PAHs and PM_2.5_ in ambient air may affect the immune development of the fetus through changes in the distributions of cord blood lymphocytes, resulting in the increased percentage of CD3^+^ and CD4^+^ lymphocytes while the decreased percentage of CD19^+^ and NK cells during early gestation [198]. In addition, long-term and short-term exposure to high concentrations of PM_2.5_, NO_2_ and CO was correlated with changes in Foxp3 differential methylated regions [199].

### The relevant mechanisms

Exogenous substances destroy the normal immune defense, including the damage of macrophages [200], the increase of cell permeability, the change of T cell populations [201] and the injury of natural killer (NK) cell reaction [182]. Exposure to PM is known to decrease the viability of macrophages [202]. It has been identified in both human and animal models that the corresponding activation of adaptive responses and cytokine production may be impaired after PM exposure, thereby increasing the susceptibility to infections [66]. Studies have demonstrated that PM_2.5_ contributes to the imbalance between the different T cell populations. In general, primary T helper type 1 (Th1) cells are designated for protection against infectious agents, while the portion of T cells called T-regs are pointed toward immunosuppression [203]. Moreover, increased production of T-regs and suppressed generation of new Th1 have been observed following PM_2.5_ exposure [204]. Furthermore, other in vitro studies have pointed out more complex effects of PM on dendritic cells (DCs) and lymphocytes. In studies with naive T cells, GM-CSF-stimulated DCs enhanced the proliferation of naive CD4 T cells in the mixed leukocyte reaction (MLR) after urban PM treatment but reduced the Th1 cell proportion [205]. Subsequent research of naive CD8 T cells in MLR showed that PM-stimulated DCs enhanced the IFN-γ production response of CD8 T cells, which indicated that PM-stimulated DCs have different immunomodulatory effects on CD4 and CD8 T cells [206]. PM is well known to cause damage, disease and death by inducing oxidative stress and the reduction of endogenous antioxidants [63]. A study has shown that exposure to PM led to oxidative damage and redox imbalance in the lungs of neonatal mice, inhibiting adaptive immune response by inducing increased regulatory T cells (Tregs), which in turn enhanced the severity of influenza virus infection in neonatal mice [201]. In general, oxidative stress is caused by a combination of organic compounds and heavy metal components of PM. Organic compounds, contained in PM or formed during the cellular metabolism of PM, can provide electrons to O_2_ to form superoxide radicals. Transition metals can also form superoxide and hydrogen peroxide by providing electrons and can directly consume the endogenous thiol antioxidant [207]. PM carries complex organic PAH molecules that can induce oxidative stress, which plays an important role in NF-κB and AP-1 signaling and gene transcription containing the antioxidant response element (ARE) promoter. In addition, the binding of PAH ligands mediated the production of more cytotoxic/genotoxic products via triggering nuclear translocation and inducing exogenous metabolic enzymes such as CYP1A1 and CYP1B1. Indeed, PAHs specifically within DEPs are thought to be the primary cause of genotoxicity, suggesting that the severity of the DNA damage caused by PM depends on the levels of PAHs [208]. Furthermore, it has been found that AHR is a special mechanism of cells for sensing PAH, which can interact with a range of antioxidant and inflammatory transcription factors such as NF-κB, Nrf2, STAT1 and RORγt. Thus, PAHs may disrupt barrier homeostasis and the production of transcription factors through AhR-ligand dysregulation [209].

The mechanism and characteristics of immunotoxicity caused by other air pollutants are similar to those of PM. Through in vitro studies, Devalia et al. found that the exposure of primary human bronchial epithelial cells (HBECs) to 400 ppb NO_2_ for 6 h resulted in significant secretion of TNF-α, CXCL8 and GM-CSF [207]. In addition, NO_2_ elevated the release of Th2 related cytokines in healthy bronchial epithelium, and increased the expression of ICAM-1, which in turn persistently stimulated neutrophilia, increasing the susceptibility to respiratory viruses [210]. Similarly, ozone can stimulate epithelial cells to produce pro-inflammatory factors such as TNF-α, GM-CSF and CXCL8 [201]. A population study has reported that higher levels of fluoride and ambient SO_2_ exposure were correlated with decreased blood eosinophils and increased bronchial hyperresponsiveness in children. Similarly, disruption of epithelial homeostasis and non-allergic eosinophilic nasal inflammation were observed in mice exposed to PM_2.5_ over 16 weeks, suggesting that long-term exposure to PM_2.5_ is able to result in eosinophilic inflammation [211].

Therefore, PM can lead to immunotoxicity through changes of immune microenvironment, developmental disorders of immune system and inflammatory infiltration, thus reducing the resistance to viral infection and external harmful stimuli. Inflammation, oxidative stress, ER stress and apoptosis are involved in impaired immune system health induced by PM. However, in the development of immune system injury caused by PM, how immune cells, inflammatory cells and various functional cells regulate and influence each other, and what specific role the involved signal pathways and key molecules play in this process have not been fully clarified at present, which needs further research.

## The effects and mechanisms of PM on reproductive system

### Mechanisms of male reproductive toxicity of PM

Exposure to PM_2.5_ or other environmental factors can interfere with multiple links of reproductive development. On the one hand, male reproductive development is extremely sensitive to environmental pollutants; on the other hand, PM has a wide and far-reaching impact on the reproductive system [212, 213]. In an epidemiological study of 327 men in Poland, PM was associated with abnormal sperm morphology and decreased testosterone levels. In addition, exposure to PM_2.5_ increased the proportion of chromatin immature cells [214]. Jurewicz et al. investigated a potential link between PM and sperm aneuploidy. Multivariate analysis showed that PM_2.5_ was significantly correlated with Y chromosome disomy, sex chromosome disomy and chromosome 21 disomy [215]. Studies have evaluated the sperm quality of college students living in different environments and found that college students living in environments with higher PM_2.5_ concentrations have higher semen malondialdehyde (MDA) levels. At the same time, it was also found that exposure to higher concentrations of PM would interfere with the sperm mitochondrial DNA (mtDNA) replication process (KE 177), destroying the integrity of mtDNA. These findings indicate that PM may cause oxidative stress (KE 1392) in sperm, producing ROS (KE 1115) that negatively affect mtDNA [216]. This is the first population study to directly report a potential association between ambient air pollution and increased levels of markers of oxidative damage in sperm. A recent cohort study showed a strong negative correlation between PM_2.5_ and sperm count and volume [217]. In addition, PM_2.5_ and severe acute respiratory syndrome coronavirus type 2 (SARS-COV-2) have a synergistic effect on inducing reproductive system damage [218]. The results of the above population-based studies have all demonstrated that PM can adversely affect the male reproductive system, but the specific mechanism has not been determined.

Some toxicological animal experiments have also studied the association between PM and reproductive system. Studies have found that exposure to PM_2.5_ can induce abnormal testicular tissue structure in SD rats, with an increased testicular tissue apoptosis index and the incidence of sperm malformation, resulting in reduced sperm density and sperm survival rate, as well as a decreased number of spermatogenic cells and mature sperm. At the same time, this study confirmed that ER stress (KE 1815) and autophagy (KE 1945) signaling pathways are the key mechanisms of PM_2.5_ induced reproductive toxicity [219]. Sperm motility analysis is a necessary test to evaluate male fertility. Researchers assessed the quality of sperm motility in mouse models exposed to high concentrations of PM_2.5_ and found a negative correlation between sperm motility and PM_2.5_. In addition, the study also suggested that PM_2.5_ is associated with decreased sperm quality (KE 520) and testicular damage depending on the NALP3-mediated inflammatory pathway (KE 151) [220]. The decrease of sperm number caused by PM_2.5_ may be due to the abnormal function of spermatogenesis (KE 1758). Exposure to PM_2.5_ may inhibit the hypothalamic-pituitary-gonad axis by inducing hypothalamic inflammation and then down-regulating the expression of testosterone synthetase and affecting the generation of mature sperm [221]. A recent animal experiment reported that PM_2.5_ produces ROS by activating the PI3K/Akt signaling pathway, thus damaging the integrity of the blood-testosterone barrier and the stability of the microbiota environment. Exposure to PM in male rats resulted in dysplasia of spermatogenic cells, decreased sperm quality and ultimately impaired reproductive function [222]. The above evidence further confirms that PM_2.5_ can cause damage to the male reproductive system. In conclusion, exposure to PM can reduce sperm quality and affect sperm production. The mechanism may be related to inflammation, oxidative stress and autophagy induced by PM_2.5_ [219, 220].

### Mechanisms of female reproductive toxicity of PM

PM not only damages the male reproductive system but also affects the normal reproductive function of females [223]. A cohort study of women treated for infertility explored the link between air pollution and biomarkers of ovarian aging. High PM_2.5_ was inversely associated with sinus follicle count, a recognized indicator of ovarian reserve, suggesting that PM_2.5_ may induce ovarian aging. The results also showed that PM_2.5_ had a more significant effect on the number of sinus follicles in women with infertility and abnormal menstrual cycles [224]. Using a mouse model, Ogliari et al. found that long-term exposure to PM_2.5_ negatively affected the future reproductive potential of female mice. Exposure to PM_2.5_ reduces the pool of primary follicles, which are more sensitive to PM_2.5_, especially during the prenatal and postnatal stages [225]. In addition, the number of oocytes in mice exposed to high concentrations of PM_2.5_ decreased and the degradation rate increased, ultimately leading to the decline of the quality of oocytes. The study found that ROS level in the exposed control was significantly increased, suggesting that PM may stimulate ROS production and promote mitochondrial oxidative stress, leading to oocyte damage [226]. Using RNA sequencing, the differentially expressed genes induced by PM_2.5_ were mainly enriched in the pathways of ROS and oxidative phosphorylation of ovarian steroids. Meanwhile, the results of systemic exposure of female C57BL/6 J mice for 4 months showed that chronic PM_2.5_ exposure could lead to ovarian dysfunction, including impaired reproductive function, endocrine disorders and decreased ovarian reserve capacity, of which the main mechanism is the triggering of ovarian apoptosis through the NF-κB/IL-6 signaling pathway and the associated oxidative phosphorylation pathway of it [227]. Benzo[a] Pyrene (BaP) is a common component of PM_2.5_. Studies have found that exposure to BaP can significantly increase ROS levels in mouse oocytes and induce early apoptosis of oocytes [228]. BaP can also lead to a low maturation rate of oocytes, abnormal meiosis, impaired mitochondrial functions and early apoptosis in progenitor mice. These findings suggest that exposure of parental mice to air pollutants has adverse effects on the oocytes of both themselves and their offspring [229]. Recent studies have assessed the effects of several drugs and phytochemicals on reproductive toxicity in females exposed to PM_2.5_ and confirmed that aspirin, vitamin C, vitamin E, ozone and resveratrol glycoside had certain protective effects on reproductive system, which provided potential strategies for the treatment of reproductive system diseases related to air pollution [227, 230].

In a word, PM may promote redox disorder of the female reproductive system, which can lead to abnormal growth and development of oocytes (KE 1837), premature senescence of ovary, and ultimately affect normal reproductive function (KE 363). However, the specific mechanism of PM_2.5_ toxicity to reproductive system has not been determined and further research is still needed.

## Conclusion and perspective

In summary, based on published literature and AOP Wiki, the linkages between disturbances caused by PM from molecule, cell, organ to individual and population were organized and synthesized. And the molecular mechanisms by which PM triggers adverse health effects in various systems were revealed. PM not only impairs the respiratory system and cardiovascular system, leading to changes in cardiopulmonary structural and function decline, but also disrupts intestinal homeostasis, induces chronic liver injury, affects the health of the digestive tract system and causes serious injury to various organs by inducing immune toxicity. Related reports also pointed out that PM can reduce sperm quality, affect spermatogenesis and cause abnormal growth and development of oocytes, leading to adverse birth outcomes and affecting normal reproductive function. Besides, a large number of studies have reported the association of PM with neurodevelopmental disorders and neurodegenerative disease characteristics. Oxidative stress, inflammation, autophagy and apoptosis are considered the main mechanisms causing harmful effects of PM. PM can lead to oxidative stress and oxidative damage, as well as induce local and systemic inflammatory responses. At the same time, the mutagenic and DNA damage effects of PM will cause alterations in gene expression profiles, which affect ER stress, autophagy, apoptosis, metabolic dysfunction, gene mutation and a series of cell physiological and biochemical processes. This will change the normal physiological function of cells, resulting in tissue cell damage. Moreover, recent studies have identified several emerging mechanisms involved in the toxicity of PM, including pyroptosis, ferroptosis and epigenetic modifications. This review would provide evidence for the screening of early effect markers of PM for the early risk assessment, as well as offer clues for preventing the harmful effects of PM and identifying the potential intervention targets. Furthermore, the systematic review of molecular insights into the mechanisms between PM and health effects is of great significance for adopting a comprehensive prevention and control strategy through the improvement of the environment and air quality to effectively reduce the morbidity and mortality of various systems diseases caused by PM.

However, there are still many molecular mechanisms that need to be explored in the complex signaling pathways of adverse health effects caused by PM. It is worth noting that PM still threatens public health, even at very low levels. Additionally, the recommended AQG levels of PM were downregulated in the latest Global Air Quality Guidelines released by WHO, which reminds us that research on deeper and more complex underlying mechanisms that contribute to the health effects of PM at low concentrations of exposure still needs to be conducted. In-depth exploration on the health effects and biological mechanisms of PM can provide the theoretical basis for the early prevention and biomarker screening of air pollution-related diseases. In addition, since the chemical composition and physicochemical properties of PM change with space and time, the real environmental pollution exists in the form of a mixture, the health impact of which is the embodiment of the combined toxicity of the pollution components. Therefore, it is equally crucial to study the harmful effects and molecular mechanisms of PM and to evaluate the exposure to various pollutants and their combined toxicity under the synergistic effect.

## Data Availability

Databases/repositories and materials is not applicable in this review.

## Abbreviations

ADHD: Attention deficit hyperactivity disorder
AHR: Aryl hydrocarbon receptor
AMPK: AMP-activated protein kinase
AO: Adverse outcome
AOP: Adverse outcome pathway
APCs: Antigen-presenting cells
AQG: Air quality guideline
ARE: Antioxidant response element
ASD: Autism spectrum disorder
BaP: Benzo[a] Pyrene
ceRNA: Competing endogenous RNAs
CO: Carbon monoxide
COPD: Chronic obstructive pulmonary disease
CRP: C-reactive protein
CVD: Cardiovascular disease
DALYs: Disability adjusted life years
DCs: Dendritic cells
EC: Elemental carbon
ER: Endoplasmic reticulum
FEV1.0: Forced expiratory volume per second
GBD: Global burden of disease
GSH: Glutathione
H3K9ac: Acetylated histone 3 lysine 9
HAEC: Human aortic endothelial cells
HBECs: Human bronchial epithelial cells
hsCRP: High-sensitivity C-reactive protein
IHD: Ischemic heart disease
IL-6: Interleukin-6
IL-8: Interleukin-8
JNK: C-Jun N-terminal kinase
KEs: Key events
LK-MPS: Liver and kidney microphysiological system
MCP-1: Monocyte chemoattractant protein 1
MIE: Molecular initiating event
MLR: Mixed leukocyte reaction
MMEF: Maximum mid-expiratory flow
Mn: Manganese
mtDNA: Mitochondrial DNA
NK: Natural killer
NO_2_: Nitrogen dioxide
MPCs: Myeloid progenitor cells
NOS_2_: Nitric oxide synthase 2
Nox: Nitrogen oxides
O_3_: Ozone
OVA: Ovalbumin
PEFR: Peak expiratory flow rate
PAH: Polycyclic aromatic hydrocarbons
PEFR%: The percentage of peak expiratory flow out of the overall expiratory flow volume
PINK1: PTEN-induced kinase 1
PM: Particulate matter
PM_10_: Inhalable particles
PM_2.5_: Fine particulate matter
REMS: Reactive electrophilic metabolites
ROS: Reactive oxygen species
SARS-COV-2: Severe acute respiratory syndrome coronavirus type 2
SST: Somatostatin
Th1: T helper type 1
TNF-α: Tumor necrosis factor-α
TRPA1: Transient receptor potential cation channel A1
UFPs: Ultrafine particles
VEGFR2: Vascular endothelial growth factor receptor 2
VOCs: Volatile organic compounds
